# *Candida albicans* Horizontal Transmission in COVID-19 Patients Hospitalized in Intensive Care Unit

**DOI:** 10.3390/jof10120864

**Published:** 2024-12-13

**Authors:** Magdalena Skóra, Katharina Rosam, Magdalena Namysł, Anna Sepioło, Mateusz Gajda, Justyna Jędras, Paweł Krzyściak, Joanna Zorska, Jerzy Wordliczek, Piotr B. Heczko, Reinhard Würzner, Michaela Lackner, Jadwiga Wójkowska-Mach

**Affiliations:** 1 Chair of Microbiology, Faculty of Medicine, Jagiellonian University Medical College, Czysta 18 Street, 31-121 Krakow, Poland; mateusz14.gajda@uj.edu.pl (M.G.); pawel.krzysciak@uj.edu.pl (P.K.); piotr.heczko@uj.edu.pl (P.B.H.); jadwiga.wojkowska-mach@uj.edu.pl (J.W.-M.); 2 Institute for Hygiene and Medical Microbiology, Medical University of Innsbruck, Schöpfstraße 41, 6020 Innsbruck, Austria; katharina.rosam@i-med.ac.at (K.R.); michaela.lackner@i-med.ac.at (M.L.); 3 Department of Microbiology, University Hospital in Krakow, Jakubowskiego Street 2, 30-688 Krakow, Poland; mnamysl@su.krakow.pl (M.N.); asepiolo@su.krakow.pl (A.S.); 4 Department of Microbiology, Faculty of Pharmacy, Jagiellonian University Medical College, Medyczna Street 9, 30-688 Krakow, Poland; 5 Hematology Clinic, University Hospital in Krakow, Jakubowskiego Street 2, 30-688 Krakow, Poland; jbjedras@gmail.com; 6 Center for Innovative Medical Education, Jagiellonian University Medical College, Medyczna 7 Street, 30-688 Krakow, Poland; joanna.zorska@uj.edu.pl; 7 Intensive Care Unit, University Hospital in Krakow, Macieja Jakubowskiego 2 Street, 30-688 Krakow, Poland; 8 Interdisciplinary Intensive Care Clinic, Jagiellonian University Medical College, Macieja Jakubowskiego 2 Street, 30-688 Krakow, Poland; j.wordliczek@uj.edu.pl

**Keywords:** *Candida albicans*, horizontal transmission, outbreak, intensive care unit, SARS-CoV-2, molecular epidemiology, COVID-19 pandemic, genotyping

## Abstract

Background: Invasive candidiasis is a predominant mycosis in hospitalized patients, and *Candida albicans* is the species most often responsible for this infection. Most candidiasis cases originate from endogenous mycobiota; therefore, *Candida* strains can easily be transferred among hospital patients and personnel. The aim of this study was to assess the possible horizontal transmission of *C. albicans* in patients with severe COVID-19 infection requiring hospitalization in the intensive care unit. Methods: In total, 59 *C. albicans* strains from 36 patients were collected from blood and lower-respiratory samples. The strains were genotyped using the RAPD method with the OPA-18 primer (5′-AGCTGACCGT-3′). Antifungal susceptibility testing was performed for amphotericin B (AMB), fluconazole (FCZ), voriconazole (VCZ), and anidulafungin (ANF) using the EUCAST method. Results: *C. albicans* strains were separated into 13 different groups according to their RAPD pattern. Two predominant clonal clusters of 17 strains isolated from 12 patients and 12 strains from 7 patients were identified, followed by clusters with 6, 4, and 8 strains isolated from 5, 4, and 3 patients, respectively. *C. albicans* strains were sensitive to AMB, FCZ, VCZ, and ANF, and antifungal susceptibility profiles were similar in all genetic clusters. Conclusions: Our study indicates that *C. albicans* strains can spread horizontally. The routes of transmission for strains in the ward have not been explained due to there being insufficient data. The transmission could have been caused by the unintentional spread of pathogens by medical personnel.

## 1. Introduction

*Candida* is a predominant human fungal pathogen that causes a wide spectrum of infections, ranging from mucocutaneous to deep-seated. The species *Candida albicans* is still the most frequently isolated from clinical samples and a leading cause of candidiasis; however, an increase in the clinical importance of other species, including those intrinsically less susceptible or resistant to antifungal drugs, has been observed over many years [1,2]. It is estimated that non-*albicans* species may constitute more than 50% of the etiological factors of candidemia [3,4]. In the last two decades, *Candida auris* in particular has attracted particular attention and concern. It is known for its multidrug resistance, resistance to disinfectants, and ability to cause healthcare-associated outbreaks due to its long survival period in the environment and the possibility of horizontal transmission by, e.g., healthcare workers [5].

The source of infection, regardless of the form of candidiasis and its severity, is usually the patient’s natural mycobiota colonizing the skin and mucous membranes. Disturbances to immune and hormonal homeostasis and other components of the microbiota, as well as an impaired skin barrier and gastrointestinal mucous membrane breakage, may lead to the intensive colonization of endogenous *Candida* spp. and their translocation to other tissues, including physiologically sterile sites [2]. Life-threatening invasive infections mainly affect patients undergoing immunosuppressive therapy, those with long-term use of corticosteroids or antibiotics, and those with long-term vascular catheters. Critically ill patients in intensive care units (ICUs) are at high risk for developing invasive candidiasis due to the accumulation of multiple predisposing factors [6,7,8,9]. Thus, the diagnosis of infection in this group of patients is difficult and complex. These patients often do not meet the criteria for an invasive fungal disease according to guidelines developed by the European Organization for Research and Treatment of Cancer (EORTC) and the Mycoses Study Group Education and Research Consortium (MSGERC) [10]. Therefore, they require separate, thorough diagnostics and the individual interpretation of test results [11,12,13].

During the global COVID-19 pandemic, it was observed that infection with, and a severe course of, the SARS-CoV-2 virus, leading to the subsequent need for intensive hospital care, also represent a factor favoring fungal infections. In COVID-19 patients, invasive candidiasis was among the most commonly reported mycoses, along with pulmonary aspergillosis and mucormycosis [14,15]. The increased susceptibility of COVID-19 patients to fungal infections and the higher incidence of candidemia in critically ill COVID-19 patients compared to non-COVID-19 patients are due to the effects of the virus dysregulating the host’s immune system and the medical procedures for treating coronavirus disease. These factors, among others, could lead to a reduced ability to defend against fungal pathogens [14,16]. In Poland, the COVID-19 pandemic has had a significant impact on the health service and medical care. Temporary units and wards were created to treat patients requiring hospitalization due to coronavirus disease. In particular, in the first period of the pandemic, the organization of medical care was quite chaotic, and access to various procedures and personal protective equipment (PPE) for healthcare workers was difficult. These factors influenced the quality of medical care and the comfort of work for medical staff [17]. Newly introduced procedures, limitations in resources, and the discomfort associated with the long-term use of PEE could have intensified the horizontal transmission of microorganisms in the hospital environment and increased the risk of infections with hospital strains. Horizontal transfer between patients admitted in COVID-19 units was described for bacterial pathogens [18,19,20]. *C. albicans* and other *Candida* species have been reported to cause nosocomial outbreaks in the past, although before the era of *C. auris*, they were considered rather rare [21,22,23]. Therefore, the question arose as to whether, during the COVID-19 pandemic, there was horizontal transmission of the most common *Candida* species—*C. albicans*—in wards where patients with severe coronavirus disease were hospitalized.

### Objective

The current publication aimed to assess the possible horizontal transmission of *C. albicans* in COVID-19-positive ICU patients during the pandemic period. Due to the retrospective nature of this analysis and insufficient clinical data, this study did not examine candidiasis and the distinction between *C. albicans* infection and colonization, but only strain transmission between patients staying on the same ward. The study included only strains deposited during routine microbiological diagnostics performed during the patients’ hospitalization. Due to the sanitary rules introduced in the COVID-19 pandemic, it was not possible to carry out additional tests, and no environmental or medical personnel studies were performed. The current analysis was limited to the species *C. albicans*, which turned out to be the predominant *Candida* species from clinical materials. Therefore, *C. albicans* isolates were genotyped, and their antifungal susceptibility profiles were compared.

## 2. Materials and Methods

### 2.1. Study Setting and Patient Population

The study covers the period between 1 May 2021 and 31 January 2022, during which 1614 patients (18,810 patient days, pds) with COVID-19, confirmed by SARS-CoV-2 PCR tests (COBAS 6800, Roche, Basel, Switzerland or in the CITO mode GeneXpert System, Cepheid, Sunnyvale, CA, USA), were hospitalized in the COVID-19-dedicated ICU of University Hospital in Krakow, Poland. Laboratory tests were performed depending on the patient’s clinical condition.

### 2.2. Candida Detection and Isolation

Classic culture methods and serological tests were used to detect *Candida* spp. in clinical samples. In the analyzed period, mannan and anti-mannan antibody-detecting assays (Platelia Candida Ag Plus and Platelia *Candida* Ab Plus, Bio-Rad, Marnes-la-Coquette, France) were performed in 22 and 15 patients, with 28 and 17 tests, respectively. Beta-D-glucan (Fungitell, Associates of Cape Cod, Inc., East Falmouth, MA, USA) was tested in 37 patients with 51 tests. All serological tests were carried out in accordance with the manufacturers’ instructions. Altogether, 238 patients had multiple blood cultures (BacT/ALERT 3D 480, bioMerieux, Marcy-l’Etoile, France; BacT/ALERT VIRTUO CLINIC, bioMerieux, Marcy-l’Etoile, France) (1047 cultures in total), and 214 underwent fungal cultures of materials collected from the lower respiratory tract (LRT) (405 tests in total) including bronchoalveolar lavage (BAL) and other materials (bronchial lavage, tracheal aspirate, and secretion from the bronchial tree), called, for the purposes of this publication, non-bronchoscopic lavage (NBL). *Candida* strains were isolated on Sabouraud glucose agar with gentamicin and chloramphenicol (OXOID) at 25 °C and 35 °C. Species identification was performed with matrix-associated laser desorption ionization–time-of-flight mass spectrometry (MALDI-TOF MS) VITEK^®^MS (bioMerieux, Marcy-l’Etoile, France) (ver. V3.2). The strains were stored at minus 70 °C for further research. Before genotyping the species, identification was confirmed via Sanger sequencing, using ITS primers.

### 2.3. Candida Albicans Genotyping

Genomic DNA was extracted from freshly grown colonies on Sabouraud glucose agar with cetyltrimethylammonium bromide (CTAB) according to the method described by Lackner et al. [24]. The strains were genotyped using the random amplified polymorphic DNA (RAPD) method with the OPA-18 primer (5′-AGCTGACCGT-3′). The PCR mixture contained 12.5 µL mastermix (Kappa2G Fast Hotstart, Merck KG, Darmstadt, Germany), 0.5 µM primer, and 30 ng of DNA template in a total reaction volume of 25 µL. The procedure included 38 cycles of 60 s at 94 °C and 60 s at 36 °C and two minutes at 72 °C according to Bautista-Munoz et al. 2003 [25]. The samples were separated on a 1.2% agarose gel (Biozym Scientific GmbH, Hessisch Oldendorf, Germany) at 170 V for 90 min. The band patterns were compared visually in sets; a difference of ≥3 was considered as indicating different genotypes.

### 2.4. Candida Albicans Antifungal Susceptibility Testing

The antifungal susceptibility testing (AFST) of *C. albicans* isolates was performed according to the European Committee on Antimicrobial Susceptibility Testing (EUCAST) method for yeasts [26]. Minimal inhibitory concentration (MIC) values were obtained for the amphotericin B (AMB) (Pol-Aura, Morąg, Poland), fluconazole (FCZ) (Pol-Aura, Poland), voriconazole (VCZ) (Pol-Aura, Poland), and anidulafungin (ANF) (Merck, Darmstadt, Germany). RPMI 1640 medium with L-glutamine and without sodium bicarbonate (Merck, Germany) supplemented with 2% glucose (Chempur, Piekary Śląskie, Poland) and buffered to pH 7 with 4-morpholinepropanesulfonic acid (MOPS; 0.165 mol/L, Fluorochem, Pune, India) was used as a culture medium. Dimethyl sulfoxide (DMSO) (Chempur, Poland) served as a solvent and diluent for drugs. The studies were performed using flat-bottom polystyrene 96-well microdilution plates (Nest, Wuxi, China). The results were read with a microdilution plate reader (Tecan Sunrise, Männedorf, Switzerland), using a wavelength of 530 nm, and interpreted according to the breakpoints determined by the EUCAST [27].

## 3. Results

During the analyzed period, 104 *Candida* strains from 53 COVID-19 patients hospitalized in the temporary COVID-19-dedicated ICU of The University Hospital in Krakow, Poland, were isolated from blood samples (11 strains) and LRT samples (93 strains). The isolated species included *C. albicans* (56.73%), *C. glabrata* (15.38%), *C. tropicalis* (12.5%), *C. dubliniensis* (9.62%), *C. inconspicua* (2.88%), *C. parapsilosis* (1.92%), and *C. lusitaniae* (0.96%). *C. auris* was not detected.

**Figure 1:**
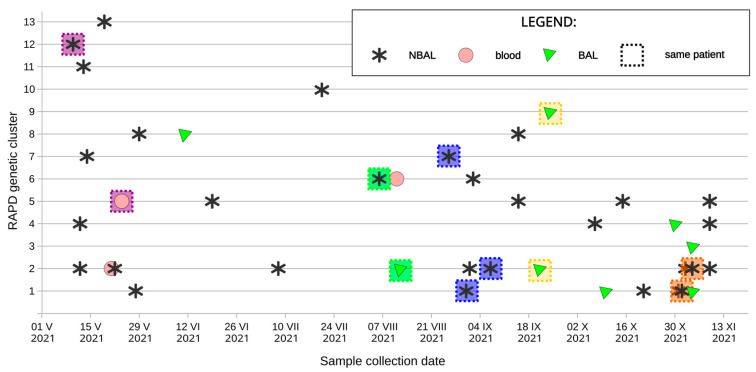
Isolation of *Candida albicans* strains belonging to different genotypes during the analyzed period. A dashed frame of the same color (blue or yellow or green or orange or purple) indicates strains isolated from the same patient. RAPD—random amplified polymorphic DNA, BAL—bronchoalveolar lavage, NBAL—non-bronchoscopic lavage (samples from lower respiratory tract other than bronchoalveolar lavage). In the case of the isolation of several *C. albicans* strains belonging to the same genotype from one patient (e.g., patient no. 28), only the strain isolated earliest is included in this Figure.

In total, 59 *C. albicans* strains (4 blood isolates and 55 LRT isolates) from 36 patients were genotyped, and antifungal drug susceptibility profiles were compared (Table 1, Table 2 and Table 3). One strain did not survive the freezing procedure (strain no 73, Table 1) and was not included in the study. Patient characteristics and data on co-infection with other *Candida* species are included in Table 1. The OPA-18 primer gave RAPD patterns of 3->10 bands (Supplementary Information Figure S1). *C. albicans* strains were separated into 13 different groups according to their RAPD pattern (Table 2, Figure 1). The biggest group forming one clonal cluster (cluster no. 2) was found to contain 17 *C. albicans* strains and was linked to 12 patients. Another big *C. albicans* cluster (cluster no. 1) was found to contain 12 *C. albicans* strains from seven patients followed by clusters no. 5, no. 4, and no. 6, with 6, 4, and 8 strains isolated from five, four, and three patients, respectively. All other clusters were minor, with 1–4 isolates per RAPD pattern (Table 2).

All *C. albicans* strains were sensitive to AMB, FCZ, VCZ, and ANF with MIC ranges of 0.062–0.25 mg/L, ≤0.125–0.5 mg/L, ≤0.008–0.031 mg/L, and ≤0.008–0.031 mg/L, respectively. Antifungal susceptibility profiles were similar in all genetic clusters (Table 3, Supplementary Information Table S1). Antifungal treatment was initiated in 10 out of 36 patients (28%) (Table 1 and Table 2). In toral, 26 patients died (72%), including 7 (70%) who had received antifungal drugs. The fatality case rate for patients colonized with strains belonging to the largest clusters, i.e., no. 2, no. 1, no. 5, and no. 4, were 75%, 71%, 80%, and 75–100%, respectively (Table 2). The data for patient treatment obtained from the computer database were incomplete; for some cases, we do not have the information on the period of treatment, only the date of initiation of the therapy. In one of the two cases of invasive candidiasis confirmed by positive blood cultures (patient no. 35) for which there was complete treatment information available, treatment (3 days CAS, then 16 days FCZ) was started two days after sample collection and proved effective (the patient survived). In the second case (patient no. 28), the patient died despite the implementation of therapy (7 days CAS, then 5 days FCZ). Therapy was initiated 5 days after blood sample collection, which could be the reason for the lack of efficacy of the therapy, but there was a long period from the end of therapy to death (26 days), which raises doubts as to the cause of death. No further blood cultures were taken, but the same genotype found in the blood was isolated 2 and 7 days after the end of antifungal therapy from NBL samples (strains no. 98 and 101) (Table 1 and Table 2). In the remaining cases in which antimycotics were used, *C. albicans* strains were isolated from LRT samples, and their clinical significance could not be determined retrospectively. In four cases (patients no. 5, 13, 20, and 25), antifungal therapy (VCZ or CAS or FCZ) was initiated 4 to 7 days after sample collection, and all cases ended in death. Additionally, patients no 5 and no 13 had co-infections with *C. glabrata*, which may also have contributed to death. In two patients (no. 12 and 18), FCZ was administered and the patients survived, but the database does not contain information on when therapy was initiated or how long it was administered. In patient no. 4, FCZ was administered two days after NBL sample collection and the patient died after 24 h of therapy, while in patient no. 22, FCZ was administered four days before NBL sample collection and the patient also died.

Some of the results were presented during the 57th Scientific Conference of the German speaking Mycological Society (DMykG), which took place in Frankfurt, Germany, on 27–29 September 2023.

Clusters no. 3 and 9–13 were represented by single *C. albicans* strains and are not included in the table. For these strains, the results of antifungal susceptibility testing are presented in Supplementary Information Table S1.

## 4. Discussion

Invasive candidiasis is a predominant fungal infection in hospitalized patients, in particular, among ICU patients, due to the accumulation of risk factors, and it is associated with high mortality [6]. Most candidiasis cases originate from the endogenous mycobiota of the patient’s skin and mucous membranes; therefore, *Candida* strains can easily be transferred among hospital patients and personnel [22,28,29,30,31,32,33]. This could be one reason for the nosocomial outbreaks of invasive *Candida* infections. The period of the COVID-19 pandemic was especially challenging for the healthcare system. Due to the large number of patients requiring hospitalization and difficulties in access to basic protective equipment for medical staff, the horizontal transmission of microorganisms occurred with greater frequency [18,19,20,34,35,36]. In this study, two predominant clonal clusters of 17 *C. albicans* strains isolated from 12 patients and 12 *C. albicans* strains from 7 patients were identified with several smaller clusters, indicating the possibility of nosocomial transmission. Patients from whom *Candida* strains were isolated were hospitalized in one ward dedicated to COVID-19 patients over a period of 9 months. During this analysis, we were not able to determine exactly how *C. albicans* strains could translocate between patients. This is due to the nature of the study, which is retrospective. Genotyping was performed only for the strains that were isolated and deposited as part of routine hospital procedures, depending on the clinical condition of the patient, which did not include the testing of environmental samples or samples from medical personnel. During the period covered by this study, special sanitary rules were introduced in Poland, and it was not possible to conduct additional research. We also had limited clinical and workflow data, lacking information on patients’ location, shared equipment, and shared medical staff. This information was not recorded in the database, so we do not have any additional evidence confirming the mode of transmission of the strains. Based on interviews with people working on the ward during that period, it appears that the severe clinical condition of patients sometimes required the use of sudden, unforeseen activities that could lead to the need to transfer patients around the ward and to accidental contacts promoting the spread and exchange of microbiota.

A major concern today in fungal healthcare-associated outbreaks is the spread of *C. auris* in the hospital environment, which have been reported worldwide [31,32,33,37,38,39,40]. We have not isolated *C. auris* strains although the incidence of COVID-19-associated candidiasis caused by this species is increasing in many hospitals, resulting in increased mortality among hospitalized patients with COVID-19 [15,41,42,43,44,45,46,47]. The first strains of *C. auris* were reported from Poland to ECDC in 2018 and 2019, but little is known about the real dissemination of this species in Polish hospitals [48]. Obviously, *C. auris* did not affect this hospital despite the COVID-19 emergence. Cases of the cross-transmission of other *Candida* species are not frequently reported but have been described before for *C. albicans*, *C. parapsilosis*, *C. tropicalis*, *C. lusitaniae*, *C. pelliculosa,* and *C. glabrata*, mostly occurring in neonatal and intensive care units [21,22,23,31,32,33,49,50]. Recently, apart from horizontal transmission and nosocomial infections of *C. auris*, emerging reports of fluconazole-resistant *C. parapsilosis* outbreaks have been described in, for example, Canada and Turkey [51,52]. Burnie et al. described a *C. albicans* outbreak of systemic candidiasis in 14 ICU patients in nine months [22]; 12 patients died. The strain responsible for this outbreak was also cultured from swabs from the oral mucous membrane and hands of medical staff, but it was not detected in the environment. This, therefore, indicates that healthcare personnel may play an important role in the transmission of *C. albicans* in hospitals.

Studying the genetic relationship between C. albicans and fungemia in the hospital can uncover the presence of endemic genotypes, which may suggest that the hands of healthcare personnel are important in the colonization and infection of *Candida* spp. [53]. Yildirim et al. (2007) found that overall, 34.1% of the hospital staff were found to harbor *Candida* spp. on their hands: 30.7% were nurses, 25.8% were resident doctors, 28.6% were laboratory workers, 84.6% were dining room personnel, and 43.3% were officers [54]. Thus, hospital workers, especially non-medical staff, should be educated in regular hand hygiene practice to prevent *Candida* colonization. Moreover, *Candida* can also colonize and persist in the hospital environment. *C. auris* was recovered from lanyards, temperature probes, floors, trollies, radiators, and windowsills. *C. parapsilosis* was found on neonatal incubators and humidifiers, patient beds, nurse workstations, computers, and floors, and *C. tropicalis* was found on haemodialysis machines. All these species have been reported to cause nosocomial epidemics [21]. In order to eliminate environmental reservoirs of *Candida*, attention should be paid to the quality of the disinfection of rooms, surfaces, and reusable materials, as well as the disinfectant used, its spectrum, and proper handling.

It would be of particular interest to repeat our study in the next period to check if the presence of the persistent endemic genotypes is confirmed and to follow their genetic evolution towards antifungal resistance. So far, all *C. albicans* strains tested in this study have been susceptible to antifungal drugs independently of where they originated. This finding may indicate that these strains do not belong to known worldwide clusters of resistant strains [55]. It also suggests that the selective pressure of antifungals used to treat invasive *C. albicans* infections has not been strong enough to lead to the presence of resistant strains with increased survival in the hospital environment. One possible factor might have been the limited use of antifungal drugs in the ICU (in this analysis, they were used in 28% of patients from whom *C. albicans* was isolated) and the short duration of therapy (in our study, in 40% of treated patients, therapy lasted for up to 3 days).

In Poland and the surrounding part of Europe, the research on fungal infections is still limited. Information on the prevalence of mycosis, species identification, patients’ conditions during hospitalization and outpatient care, resistance, and genetics is sparse [56,57,58,59,60]. Fungal infections are only marginally treated, even in patients in the ICU or who are immunosuppressed. Increasing the awareness of fungal infections, as well as of the problem of horizontal transmission within hospitals, is crucial in decreasing the number of mycoses and fungal outbreaks. We hope that this study will draw more attention to fungal infections in our geographical area and will contribute to the more effective prevention, detection, and treatment of fungal infections.

## 5. Conclusions

Our study indicates that *C. albicans* can spread horizontally. The role of healthcare workers and the environment in the emergence of *C. albicans* nosocomial infections in critical areas through the unintentional spread of pathogens has not been confirmed because of limited data. The obtained results indicate the need to conduct a study covering not only hospitalized patients but also the environment and medical personnel. This will allow the confirmation of the horizontal transmission of *Candida* fungi, the determination of potential causes, and the introduction of preventive measures. It seems advisable to improve logistic and clinical data collection and banking, so that they can represent a reliable and complete source of information.

## Figures and Tables

**Table 1:** Characteristics of COVID-19 patients hospitalized in the intensive care unit with the isolation of *Candida albicans* from blood and lower respiratory tract samples.

Patient Characteristics							*C. albicans* Cultures				Other *Candida* Species Cultures (Type of Sample, Date of Collection)	Antifungal Treatment	Death
Patient No	Sex	Age [Year]	LOS [Day]	LOS/ICU [Day]	CVC	MV	Strain No	Type of Sample	Sample Collection Date	RAPD Genetic Cluster			
1	M	62	76	76	Y	Y	122/A	NBL	15 October 2021	5	*C. tropicalis* (NBL, 15 October and 19 November 2021) *	N	Y
2	M	35	9	9	Y	N	96	NBL	1 September 2021	2	N	N	N
3	M	71	32	32	Y	Y	6	NBL	12 May 2021	2	*C. glabrata*, *C. tropicalis* (NBL, 18 April 2021)	N	Y
							18	BAL	17 May 2021	2			
4	F	73	14	14	Y	Y	163	NBL	9 September 2021	2	N	Y (1-day FCZ)	Y
5	F	75	52	51	Y	Y	32	Blood	23 May 2021	2	*C. glabrata* (NBL, 12 April and 5 May 2021) *	Y (1-day VCZ)	Y
							33	Blood	21 May 2021	2			
6	M	60	13	13	Y	Y	28A	NBL	22 May 2021	2	*C. glabrata* (NBL, 22 May 2021) (urine, 22 May 2021) *	N	Y
7	M	81	2	2	Y	Y	138	NBL	2 November 2021	2	N	N	Y
8	M	71	27	27	Y	Y	23	NBL	19 May 2021	13	N	N	Y
9	F	82	18	12	Y	Y	147	BAL	4 November 2021	3	N	N	Y
10	M	69	7	2	Y	Y	67	NBL	19 June 2021	5	N	N	Y
11	F	63	23	23	Y	Y	117	BAL	8 October 2021	2	*C. lusitaniae* (NBL, 21 September and 24 September 2021) *	N	Y
							109A	BAL	21 September 2021	2			
							111B	BAL	24 September 2021	9			
12	F	73	78	77	Y	Y	77	NBL	6 August 2021	6	N	Y (FCZ)	N
							85	NBL	24 August 2021	2			
							79A	BAL	12 August 2021	2			
							91A	NBL	30 August 2021	2			
13	F	51	42	22	Y	Y	45	NBL	29 May 2021	8	*C. glabrata* (urine, 13 May and 22 May and 29 May 2021)	Y (3-day CAS)	Y
14	F	72	6	6	Y	Y	161	NBL	9 November 2021	5	N	N	Y
15	F	80	21	21	Y	Y	123	NBL	21 October 2021	1	N	N	Y
							145	NBL	3 November 2021	1			
16	F	77	46	46	Y	Y	56	BAL	11 June 2021	8	N	N	N
17	F	75	15	15	Y	Y	116	NBL	7 October 2021	4	N	N	Y
18	M	32	34	x	N	N	10B	BAL	13 May 2021	11	N	Y (FCZ)	N
19	F	66	61	40	Y	Y	73	NBL	4 July 2021	Not tested	N	N	N
							74	NBL	8 July2021	2			
20	M	60	27	9	Y	Y	108	NBL	19 September 2021	5	N	Y (VCZ)	Y
							104B	NBL	15 September 2021	5			
21	M	31	10	9	Y	Y	136	NBL	1 November 2021	1	N	N	N
							146	NBL	3 November 2021	1			
							151	BAL	4 November 2021	1			
22	M	56	27	16	Y	Y	8B	NBL	12 May 2021	4	N	Y (FCZ)	Y
23	M	65	32	22	Y	Y	102	NBL	15 September 2021	8	N	N	Y
24	M	68	18	18	Y	Y	132	BAL	30 October 2021	4	N	N	N
25	M	68	24	22	Y	Y	89	NBL	26 August 2021	7	N	Y (FCZ)	Y
							93	NBL	31 August 2021	1			
							99	NBL	7 September 2021	2			
26	F	66	23	5	Y	Y	159/A	NBL	9 November 2021	4	*C.glabrata* (NBL, urine 9 November and 13 November 2021), *C. krusei* (NBL, urine 9 Nomvember 2021) (urine, 13 November 2024), *C.dubliniensis* (blood, NBL, 13 November 2024)	N	Y
27	F	29	24	12	Y	Y	153	BAL	4 November 2021	1	N	N	N
28	M	76	54	53	Y	Y	80	NBL	13 August 2021	6	N	Y (17-day CAS) (5-day FCZ)	Y
							81	Blood	11 August 2021	6			
							86	NBL	24 August 2021	6			
							95	NBL	31 August 2021	6			
							98	NBL	4 September 2021	6			
							101	NBL	8 September 2021	6			
29	M	66	99	25	Y	Y	97	NBL	2 September 2021	6	N	N	Y
30	M	58	11	4	Y	Y	134	NBL	31 October 2021	1	N	N	Y
							150	NBL	4 November 2021	2			
31	F	71	13	11	Y	Y	14	NBL	14 May 2021	7	N	N	Y
32	M	73	38	30	Y	Y	119	BAL	10 October 2021	1	N	N	Y
							124	NBL	22 October 2021	1			
33	F	33	29	17	Y	Y	43	NBL	28 May 2021	1	N	N	Y
							57A	BAL	9 June 2021	1			
34	M	82	95	63	Y	Y	112	NBL	26 September 2021	10	N	N	N
35	M	71	144	142	Y	Y	4	NBL	10 May 2021	12	N	Y (3-day CAS), (16-day FCZ)	N
							40	Blood	24 May 2021	5			
36	M	58	13	1	Y	Y	140	NBL	2 November 2021	2	N	N	Y

CVC—central venous catheter, LOS—length of hospital stay, LOS/ICU—length of stay in the intensive care unit, MV—mechanical ventilation, NBL—non-bronchoscopic lavage (lower respiratory tract samples other than bronchoalveolar lavage), FCZ—fluconazole, VCZ—voriconazole, CAS—caspofungin, N—no, Y—yes, * *C. albicans* was also isolated from the samples.

**Table 2:** *Candida albicans* genetic clusters in random amplified polymorphic DNA (RAPD) assay.

RAPD Genetic Cluster	*C. albicans* Strain No	Gel Picture No	Type of Clinical Sample	Sample Collection Date	Patient No	Hospitalization Date	Antifungal Treatment	Death
1	43	12	BAL	28 May 2021	33	20 May 2021	no	17 June 2021
	57A	15	BAL	9 June 2021				
	93	26	BAL	31 August 2021	25	16 August 2021	fluconazole (2 September 2021)	9 September 2021
	119	41	BAL	10 October 2021	32	27 September 2021	no	3 November 2021
	124	44	NBL	22 October 2021				
	123	43	BAL	21 October 2021	15	21 October 2021	no	11 November 2021
	145	50	NBL	3 November 2021				
	134	46	BAL	31 October 2021	30	25 October 2021	no	4 November 2021
	136	47	BAL	1 November 2021	21	30 October 2021	no	no
	146	51	BAL	3 November 2021				
	151	54	BAL	4 November 2021				
	153	55	BAL	4 November 2021	27	30 October 2021	no	no
2	33	10	Blood	21 May 2021	5	05.04.2021	voriconazole (26 May 2021)	26 May 2021
	32	9	Blood	23 May 2021				
	6	2	NBL	12 May 2021	3	17.04.2021	no	19 May 2021
	18	6	BAL	17 May 2021				
	28A	8	BAL	22 May 2021	6	13 May 2021	no	26 May 2021
	74	17	NBL	8 July 2021	19	25 June 2021	no	no
	79A	19	BAL	12 August 2021	12	5 August 2021	fluconazole	no
	85	22	BAL	24 August 2021				
	91A	25	BAL	30 August 2021				
	99	31	BAL	7 September 2021	25	16 August 2021	fluconazole	9 September 2021
	96	28	NBL	1 September 2021	2	1 September 2021	no	no
	109A	36	BAL	21 September 2021	11	20 September 2021	no	13 October 2021
	117	40	BAL	8 October 2021				
	140	49	BAL	2 November 2021	36	22 October 2021	no	3 November 2021
	150	53	BAL	4 November 2021	30	25 October 2021	no	4 November 2021
	163	58	BAL	9 November 2021	4	29 October 2021	fluconazole (11 November 2021)	12 November 2021
	138	48	BAL	2 November 2021	7	1 November 2021	no	3 November 2021
3	147	52	BAL	4 November 2021	9	23 April 2021	no	11 May 2021
4	8B	3	NBL	12 May 2021	22	22 April 2021	fluconazole (8 May 2021)	18 May 2021
	116	39	BAL	7 October 2021	17	26 September 2021	no	11 October 2021
	159/A	56	BAL	9 November 2021	26	23 October 2021	no	9 November 2021
	132	45	BAL	30 October 2021	24	30 October 2021	no	no
5	40	11	Blood	24 May 2021	35	4 April 2021	caspofungin (26 May–28 May 2021), fluconazole (28 May–12 June 2021)	no
	67	16	BAL	19 June 2021	10	13 June 2021	no	19 June 2021
	104B	34	BAL	15 September 2021	20	29 August 2021	voriconazole (22 September 2021)	24 September 2021
	108	35	BAL	19 September 2021				
	122/A	42	BAL	15 October 2021	1	3 October 2021	no	18 December 2021
	161	57	BAL	9 November 2021	14	4 November 2021	no	10 November 2021
6	97	29	BAL	2 September 2021	29	14 June 2021	no	20 September 2021
	77	18	BAL	6 August 2021	12	5 August 2021	fluconazole (14–18 August 2021)	no
	81	21	Blood	11 August 2021	28	5 August 2021	caspofungin (16 August–22 August 2021), fluconazole (29 August–2 September 2021)	28 September 2021
	80	20	BAL	13 August 2021				
	86	23	BAL	24 August 2021				
	95	27	BAL	31 August 2021				
	98	30	BAL	4 September 2021				
	101	32	NBL	8 September 2021				
7	14	5	BAL	14 May 2021	31	1 May 2021	no	14 May 2021
	89	24	BAL	26 August 2021	25	16 August 2021	fluconazole (2 September 2021)	9 September 2021
8	45	13	BAL	29 May 2021	13	24 April 2021	caspofungin (2 June–4 June 2021)	4 June 2021
	56	14	BAL	11 June 2021	16	5 June 2021	no	no
	102	33	BAL	15 September 2021	23	17 August 2021	no	18 September 2021
9	111B	37	BAL	24 September 2021	11	20 September 2021	no	13 October 2021
10	112	38	BAL	26 September 2021	34	12 September 2021	no	no
11	10B	4	BAL	13 May 2021	18	30 April 2021	fluconazole	no
12	4	1	NBL	10 May 2021	35	4 April 2021	caspofungin (26–28 May 2021), fluconazole (28 May–12 June)	no
13	23	7	BAL	19 May 2021	8	1 May 2021	no	27 May 2021

BAL—bronchoalveolar lavage, NBL—non-bronchoscopic lavage (samples from lower respiratory tract other than bronchoalveolar lavage).

**Table 3:** Minimal inhibitory concentration (MIC) values [mg/L] obtained for the most numerous *Candida albicans* clusters.

*C. albicans* RAPD Cluster Number	AMB			FCZ			VCZ			ANF		
	MIC Range	MIC_50_	MIC_90_	MIC Range	MIC_50_	MIC_90_	MIC Range	MIC_50_	MIC_90_	MIC Range	MIC_50_	MIC_90_
1	0.012–0.25	0.125	0.125	≤0.125–0.25	0.25	0.25	≤0.008–0.016	≤0.008	0.016	≤0.008	≤0.008	≤0.008
2	0.06–0.25	0.125	0.125	≤0.125–0.5	0.25	0.5	≤0.008–0.031	0.016	0.03	≤0.008–0.031	≤0.008	0.016
4	0.06–0.25	0.125	0.25	0.125–0.25	0.25	0.25	≤0.008	≤0.008	≤0.008	≤0.008–0.016	≤0.008	0.016
5	0.06–0.125	0.125	0.125	≤0.125–0.5	0.25	0.5	≤0.008–0.031	≤0.008	0.03	≤0.008–0.016	≤0.008	0.016
6	0.06–0.125	0.125	0.125	≤0.125–0.25	≤0.125	0.25	≤0.008–0.016	≤0.008	0.016	≤0.008–0.016	≤0.008	0.016
7	0.125	0.125	0.125	≤0.125–0.25	≤0.125	0.25	≤0.008–0.031	≤0.008	0.03	≤0.008	≤0.008	≤0.008
8	0.125	0.125	0.125	0.25	0.25	0.25	0.016–0.031	0.016	0.03	≤0.008	≤0.008	≤0.008

AMB—amphotericin B, FCZ—fluconazole, VCZ—voriconazole, ANF—anidulafungin, MIC—minimal inhibitory concentration, MIC_50_—concentration of antifungal drug able to inhibit 50% of strains tested, MIC_90_—concentration of antifungal drug able to inhibit 90% of strains tested.

## Data Availability

The datasets generated and/or analyzed during the current study are not publicly available but will be available from the corresponding authors on reasonable request.

## Supplementary Materials

The following supporting information can be downloaded at: https://www.mdpi.com/article/10.3390/jof10120864/s1, Figure S1: A-D: RAPD patterns of *Candida albicans* strains using OPA-18 primer. The corresponding strain numbers can be found in Table 2 with the genotypes. L correlates to the DNA standard (New England Biolabs). Table S1: Amphotericin B, fluconazole, voriconazole, and anidulafungin minimal inhibitory concentrations against *Candida albicans* strains belonging to different genetic clusters.
