# Supplementary material for: Candida albicans Horizontal Transmission in COVID-19 Patients Hospitalized in Intensive Care Unit
Source: J Fungi (Basel). 2024 Dec 13;10(12):864. doi: 10.3390/jof10120864 (PMC11677274; doi:10.3390/jof10120864)
Supplement: Supplementary file 1 [file jof-10-00864-s001.zip › jof-3330826-supplementary.pdf]

Supplementary Information (SI)

Title: Candida albicans horizontal transmission in COVID-19 patients hospitalized in intensive care unit in university hospital in Poland

Authors: Magdalena Skóra, Katharina Rosam, Magdalena Namysł, Anna Sepioło, Mateusz Gajda, Justyna Jędras, Paweł Krzyściak, Joanna Zorska, Jerzy Wordliczek, Piotr B. Heczko, Reinhard Würzner, Michaela Lackner, Jadwiga Wójkowska-Mach

Journal: Journal of Fungi

Corresponding authors:

Magdalena Skóra, Chair of Microbiology, Faculty of Medicine, Jagiellonian University Medical College, Czysta 18 Street, 31-121 Krakow, Poland; ORCID 0000-0002-4043-1628, magdalena.skora@uj.edu.pl

Reinhard Würzner, Prof., Medical University of Innsbruck, Institute for Hygiene and Medical Microbiology, Schöpfstraße 41, 6020 Innsbruck, Austria; ORCID 0000-0001-6804-442X, reinhard.wuerzner@i-med.ac.at

Supplementary Information Figure S1. A-D: RAPD patterns of *C. albicans* strains using OPA-18 primer. The corresponding strain numbers can be found in Table 2 with the genotypes. L correlates to the DNA standard (New England Biolabs).

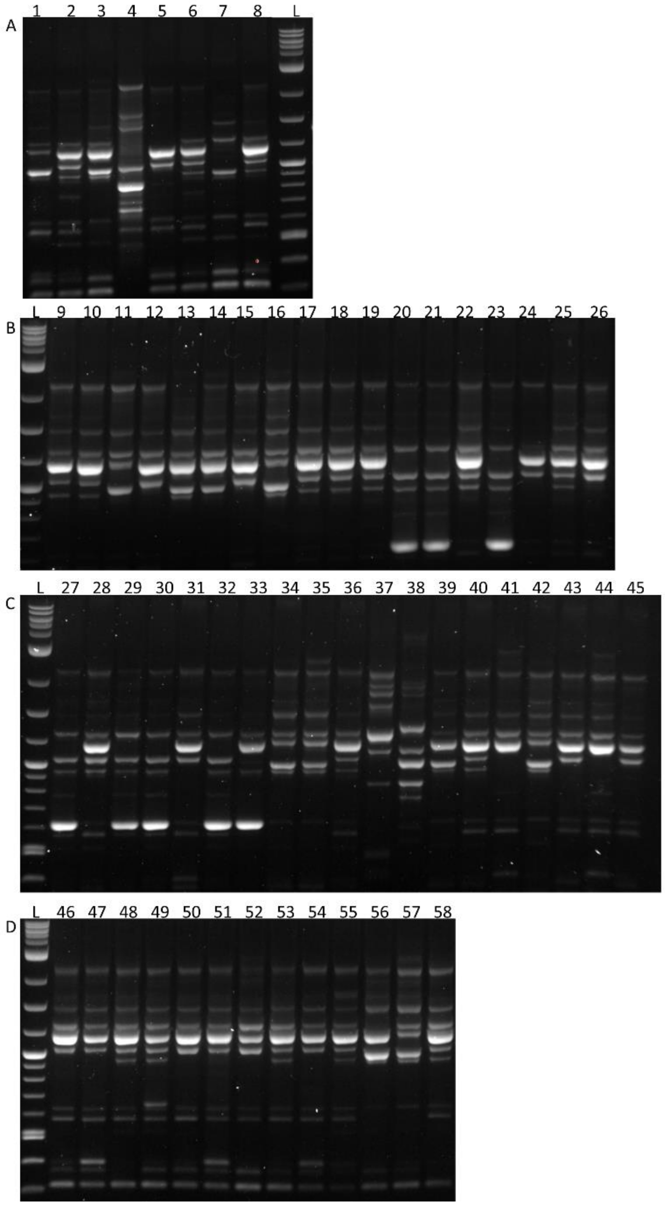

Supplementary Information Table S1. Amphotericin B, fluconazole, voriconazole, and anidulafungin minimal inhibitory concentrations against *Candida albicans* strains belonging to different genetic clusters

**Genetic cluster**	***C. albicans* strain number**	**AMB**	**FCZ**	**VCZ**	**ANF**
1	93	0.125	≤0.125	≤0.008	≤0.008
	57A	0.125	≤0.125	≤0.008	≤0.008
	124	0.012	0.25	≤0.008	≤0.008
	123	0.06	0.25	≤0.008	≤0.008
	145	0.125	0.25	≤0.008	≤0.008
	43	0.125	0.25	≤0.008	≤0.008
	119	0.25	0.25	≤0.008	≤0.008
	146	0.125	≤0.125	0.016	≤0.008
	134	0.062	0.125	0.016	≤0.008
	136	0.062	0.25	0.016	≤0.008
	151	0.125	0.25	0.016	≤0.008
	153	0.125	0.25	0.016	≤0.008
2	33	0.125	≤0.125	≤0.008	≤0.008
	32	0.125	≤0.125	≤0.008	≤0.008
	74	0.125	≤0.125	≤0.008	≤0.008
	99	0.125	≤0.125	≤0.008	≤0.008
	150	0.125	≤0.125	≤0.008	≤0.008
	96	0.125	0.25	≤0.008	≤0.008
	28A	0.125	0.25	≤0.008	≤0.008
	117	0.06	0.25	0.016	≤0.008
	18	0.125	0.25	0.016	≤0.008
	6	0.125	0.5	0.016	≤0.008
	109A	0.125	0.5	0.016	≤0.008
	140	0.062	0.25	0.031	≤0.008
	138	0.125	0.25	0.016	0.016
	79A	0.25	0.25	0.03	0.016
	91A	0.125	0.25	0.016	0.031
	163	0.125	0.25	0.016	undefined
	85	0.125	0.25	0.016	undefined
3	147	0.125	0.25	≤0.008	≤0.008
4	8B	0.125	0.125	≤0.008	≤0.008
	116	0.125	0.25	≤0.008	≤0.008

	132	0.25	0.25	≤0.008	≤0.008
	159/A	0.062	0.25	≤0.008	0.016
5	67	0.06	0.25	0.016	≤0.008
	122/A	0.125	0.25	0.016	≤0.008
	161	0.125	0.25	0.016	≤0.008
	104B	0.125	0.25	0.016	≤0.008
	108	0.125	0.5	0.031	≤0.008
	40	0.125	≤0.125	≤0.008	0.016
6	80	0.125	≤0.125	≤0.008	≤0.008
	86	0.125	≤0.125	≤0.008	≤0.008
	98	0.125	≤0.125	≤0.008	≤0.008
	95	0.06	0.25	≤0.008	≤0.008
	97	0.125	0.25	0.016	≤0.008
	77	0.06	0.25	0.016	0.016
	101	0.06	≤0.125	≤0.008	not tested
	81	0.06	0.25	≤0.008	not tested
7	89	0.125	≤0.125	≤0.008	≤0.008
	14	0.125	0.25	0.031	≤0.008
8	102	0.125	0.25	0.016	≤0.008
	45	0.125	0.25	0.031	≤0.008
	56	not tested	not tested	not tested	≤0.008
9	111B	undefined	undefined	undefined	≤0.008
10	112	0.125	0.5	0.016	≤0.008
11	10B	0.031	0.25	0.016	≤0.008
12	4	0.125	≤0.125	≤0.008	≤0.008
13	23	0.125	≤0.125	≤0.008	≤0.008

AMB – amphotericin B, FCZ – fluconazole, VCZ – voriconazole, ANF – anidulafungin
